# Correction to “Tumor‐derived *Prevotella intermedia* aggravates gastric cancer by enhancing Perilipin 3 expression”

**DOI:** 10.1111/cas.70005

**Published:** 2025-02-12

Liang W, Zhou Z, Gao Q, et al. Tumor‐derived *Prevotella intermedia* aggravates gastriccancer by enhancing Perilipin 3 expression. Cancer Sci. 2024;115:1141–1153. doi:10.1111/cas.16080.

In the version of this article initially published, the survival curve in Figure 2B was incorrectly displayed due to the incorrect follow‐up time. The correct image is shown below:

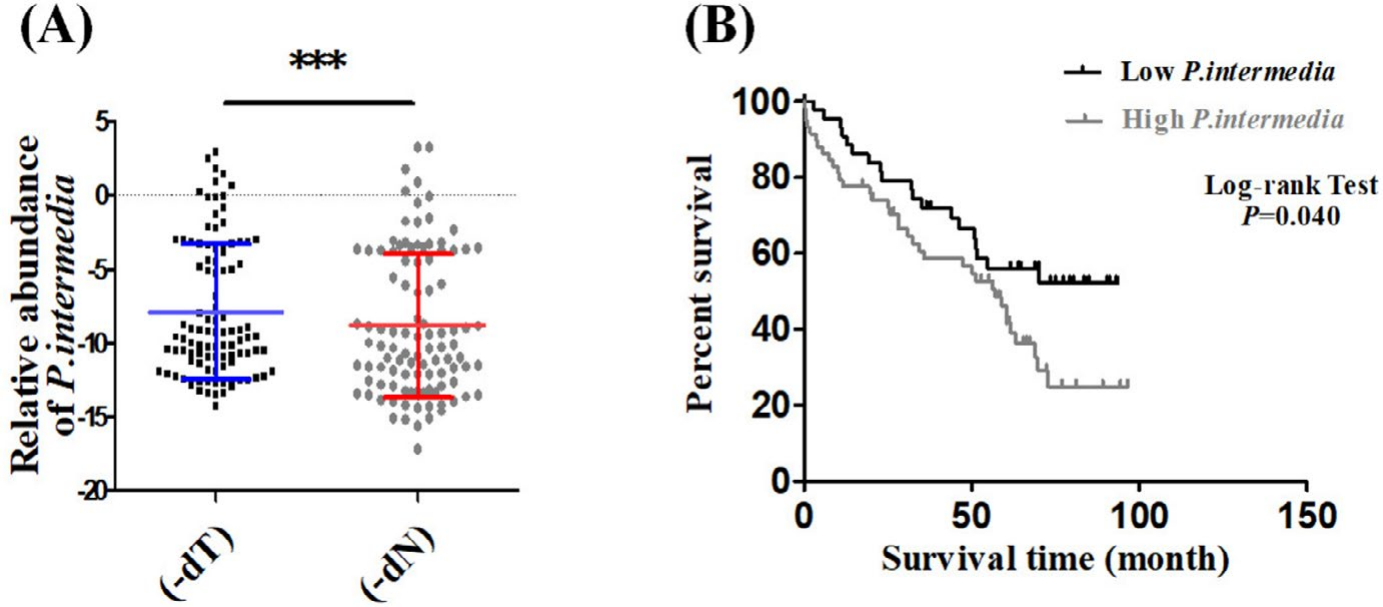

In the Abstract section, the text “The abundance of *P. intermedia* exhibited correlations with tumor differentiation (*p* = 0.006), perineural invasion (*p* = 0.004), omentum majus invasion (*p* = 0.040), and the survival duration of GC patients (*p* = 0.04**2**)”. It should instead be “The abundance of *P. intermedia* exhibited correlations with tumor differentiation (*p* = 0.006), perineural invasion (*p* = 0.004), omentum majus invasion (*p* = 0.040), and the survival duration of GC patients (*p* = 0.04**0**)”.

We apologize for this error.

